# A phenyl-thiadiazolylidene-amine derivative ejects zinc from retroviral nucleocapsid zinc fingers and inactivates HIV virions

**DOI:** 10.1186/1742-4690-9-95

**Published:** 2012-11-12

**Authors:** Thomas Vercruysse, Beata Basta, Wim Dehaen, Nicolas Humbert, Jan Balzarini, François Debaene, Sarah Sanglier-Cianférani, Christophe Pannecouque, Yves Mély, Dirk Daelemans

**Affiliations:** 1 Rega Institute for Medical Research, Laboratory for Virology and Chemotherapy, KU Leuven, Minderbroedersstraat 10, Leuven, B-3000, Belgium; 2 Laboratoire de Biophotonique et Pharmacologie, UMR 7213 du CNRS, Université de Strasbourg, Faculté de Pharmacie, 74 route du Rhin, Illkirch, 67401, France; 3 Chemistry Department, KU Leuven, Celestijnenlaan 200F, Leuven, B-3001, Belgium; 4 Laboratoire de Spectrométrie de Masse BioOrganique (LSMBO), Université de Strasbourg, IPHC, 25 rue Becquerel, Strasbourg, 67087, France; 5 CNRS, UMR7178, Strasbourg, 67037, France

**Keywords:** HIV, Nucleocapsid, Virucide, Microbicide

## Abstract

**Background:**

Sexual acquisition of the human immunodeficiency virus (HIV) through mucosal transmission may be prevented by using topically applied agents that block HIV transmission from one individual to another. Therefore, virucidal agents that inactivate HIV virions may be used as a component in topical microbicides.

**Results:**

Here, we have identified 2-methyl-3-phenyl-2*H*-[1,2,4]thiadiazol-5-ylideneamine (WDO-217) as a low-molecular-weight molecule that inactivates HIV particles. Both HIV-1 and HIV-2 virions pretreated with this compound were unable to infect permissive cells. Moreover, WDO-217 was able to inhibit infections of a wide spectrum of wild-type and drug-resistant HIV-1, including clinical isolates, HIV-2 and SIV strains. Whereas the capture of virus by DC-SIGN was unaffected by the compound, it efficiently prevented the transmission of DC-SIGN-captured virus to CD4^+^ T-lymphocytes. Interestingly, exposure of virions to WDO-217 reduced the amount of virion-associated genomic RNA as measured by real-time RT-qPCR. Further mechanism-of-action studies demonstrated that WDO-217 efficiently ejects zinc from the zinc fingers of the retroviral nucleocapsid protein NCp7 and inhibits the cTAR destabilization properties of this protein. Importantly, WDO-217 was able to eject zinc from both zinc fingers, even when NCp7 was bound to oligonucleotides, while no covalent interaction between NCp7 and WDO-217 could be observed.

**Conclusion:**

This compound is a new lead structure that can be used for the development of a new series of NCp7 zinc ejectors as candidate topical microbicide agents.

## Background

Human immunodeficiency virus type 1 (HIV-1), the causative agent of AIDS (Acquired Immune Deficiency Syndrome), still represents a serious global public health problem. Although established anti-HIV treatments are relatively effective, they are sometimes poorly tolerated, highlighting the need for further refinement of the existing antiviral drugs and the development of novel anti-HIV strategies. In this respect, the use of topical virucides would be an interesting therapeutic strategy to prevent HIV transmission. Several topical agents for preventing HIV transmission have been described, including i) agents that inactivate HIV such as detergents (e.g. Nonoxynol-9 or SAVVY®) or *p*H modifiers (e.g. BufferGel; ReProtect [1]), ii) agents that target viral replication (e.g. the reverse transcriptase inhibitors UC-781, TMC-120, tenofovir) and iii) agents that target viral entry (e.g. PRO 2000, cellulose sulfate). With the exception of tenofovir [2], most microbicide candidate drugs that have been subject of large clinical trials, have proved to be non-effective or even toxic upon long-term exposure of the vaginal environment to these products.

The HIV-1 nucleocapsid NCp7 is essential for and plays multiple roles in virus replication [3]. NCp7 contains two CCHC zinc-finger motifs and binds the viral genomic RNA in the interior of the virion. The binding of NCp7 to nucleic acids results in their condensation and protection from nuclease degradation [4-6]. Therefore, this nucleoprotein complex protects the genomic RNA ensuring viral stability. A number of classes of compounds targeting the retroviral NCp7 have been described, including, 3-nitrosobenzamide (NOBA) [7], 2,2^′^-dithiobisbenzamides (DIBA) [8], cyclic 2,2^′^-dithiobisbenzamides (e.g. SRR-SB3) [9], 1,2-dithiane-4,5-diol-1,1-dioxide [10], azadicarbonamide (ADA) [11,12], pyridinioalkanoyl thiolesters (PATEs) [13], bis-thiadiazolbenzene-1,2-diamines [14] and S-acyl-2-mercaptobenzamide thioesters (SAMTs) [15]. The latter class of compounds was recently considered for testing as topical microbicide for the prevention of HIV transmission [16]. These SAMT compounds were able to efficiently prevent vaginal transmission of SHIV upon exposure of nonhuman primates [17]. Here, we have identified a low-molecular-weight molecule, 2-methyl-3-phenyl-2*H*-[1,2,4]thiadiazol-5-ylideneamine, WDO-217, that inhibits HIV replication. Mechanism-of-action studies reveal WDO-217 as a potent NCp7 zinc ejector that directly inactivates HIV-1 and HIV-2 virions and inhibits the transmission of DC-SIGN captured virus to CD4^+^ lymphocytes. WDO-217 qualifies as a potential microbicide lead compound for further (pre)clinical studies.

## Results and discussion

### Inhibition of HIV and SIV in cell culture

**Figure 1:**
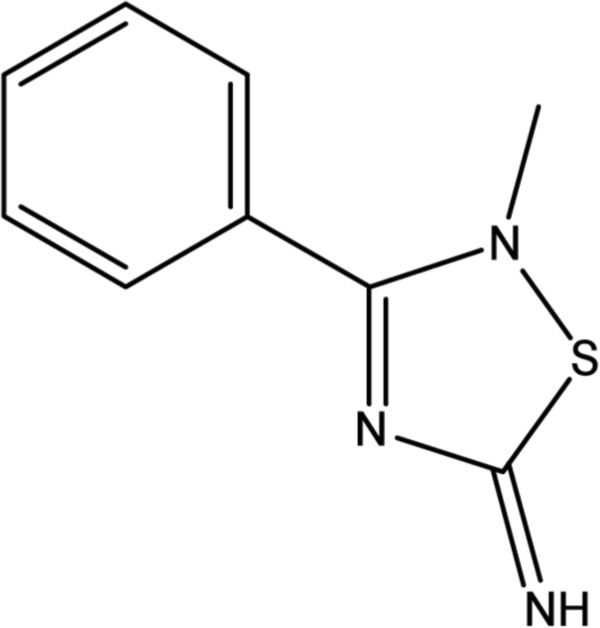
**Chemical structure of WDO-217.** Molecular weight: 191.25.

**Figure 2:**
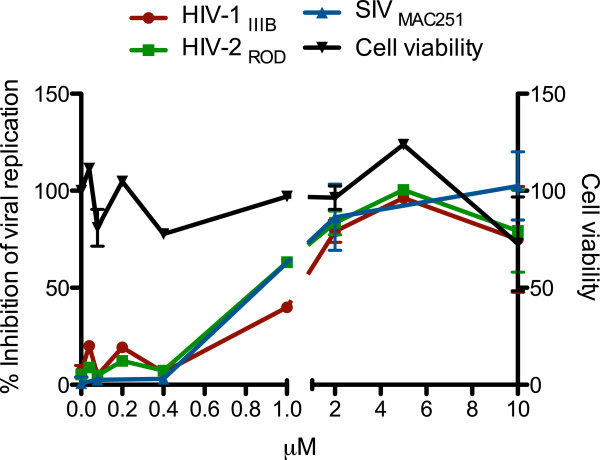
**Dose-dependent inhibitory effect of WDO-217 on the replication of HIV-1 III**_**B**_**, HIV-2 ROD, and SIV Mac251.** MT-4 T-cells were infected with the respective viruses and incubated in the presence of compound. Protection against HIV-induced cytopathic effect was monitored 5 days after infection using the MTT-assay [18]. Cell viability in the presence of compound but in the absence of virus, was measured in parallel. Results are presented as mean ± std dev from at least 2 independent experiments each in triplicate.

2-Methyl-3-phenyl-2*H*-[1,2,4]thiadiazol-5-ylideneamine (WDO-217) (Figure 1) was identified in a high-throughput screen for anti-HIV and anti-SIV activity in cell culture (Figure 2 and Table 1) [18,19]. WDO-217 was equally active against HIV-1 (III_B_) (EC_50_: 5 ± 3 μM), HIV-2 (ROD) (EC_50_: 2.3 ± 0.3 μM), and SIV (Mac251) (EC_50_: 5 ± 1 μM), while its 50% cytotoxic concentration in MT-4 cell cultures was around 72 μM resulting in an average selective index of approximately 20. Its antiretroviral activity was confirmed in human T-lymphocyte CEM cell cultures (Table 2). In contrast, the compound was inactive against the replication of viruses other than retroviruses, including CMV, HSV, VZV, VSV, RSV.

**Table 1:** Antiretroviral activity and cytotoxicity of WDO-217 in the MT-4/MTT-assay

**Compound**	**EC**_**50 **_**(μM)**			**CC**_**50 **_**(μM)**
	**HIV-1 III**_**B**_	**HIV-2 ROD**	**SIV Mac251**	
WDO-217	7.9 ± 3.3	2.3 ± 0.3	5.3 ± 1.5	72 ± 11
AZT	0.007 ± 0.001	0.005 ± 0.0008	0.016 ± 0.007	>35

Values are presented as mean ± std dev from at least 3 independent experiments.

*EC*_*50*_: 50% effective concentration, concentration of inhibitor required for 50% inhibition of viral replication.

*CC*_*50*_: 50% cytotoxic concentration, concentration of inhibitor that kills 50% of the cells.

**Table 2:** Anti-HIV-1 and -HIV-2 activity and cytostatic properties of WDO-217 in human T-lymphocyte (CEM) cells

**Compound**	**EC**_**50 **_**(μM)**		**CC**_**50 **_**(μM)**
	**HIV-1 III**_**B**_	**HIV-2 ROD**	
WDO-217	8.3 ± 1.8	15 ± 6.7	94 ± 15
AZT	0.058 ± 0.030	0.055 ± 0.020	>125

*EC*_*50*_: effective concentration or concentration required to protect CEM cells against the cytopathogenicity of HIV by 50%.

*CC*_*50*_: cytotoxic concentration or concentration compound required to reduce CEM cell proliferation by 50%.

Values are the mean ± std dev of about 2 to 3 independent experiments.

To assess the potential of WDO-217 against drug-resistant HIV-1 strains, its antiviral activity was examined against virus strains that are resistant to either the entry inhibitor dextran sulfate, the CXCR4 antagonist AMD3100, the NRTI AZT, or the NNRTI nevirapine. Therefore, Jurkat A72 cells containing LTR-GFP [20] were infected with the respective drug-resistant virus strains in the presence of the test compound. Production of Tat protein as a result of viral replication drives the integrated LTR to produce GFP. Infected cells become brightly fluorescent and can be measured by flow cytometry, providing a direct and quantitative marker for HIV-1 infection in individual live cells. Cells were harvested 3 days after infection, and the number of GFP-expressing cells was monitored. Toxicity of the compounds was assessed from an MTT-based viability assay. WDO-217 invariably retained its full anti-HIV activity against the different virus strains (Table 3) whereas dextran sulphate, AMD3100, AZT, and nevirapine markedly lost inhibitory potential against their respective resistant HIV-1 mutants. These results incited us to carry out a detailed study on the target of antiviral action of WDO-217.

**Table 3:** Antiretroviral activity and cytotoxicity of WDO-217 in the anti-HIV

**Compound**	**EC**_**50 **_**μM**							**CC**_**50 **_**μM**
	**HIV-1 III**_**B**_	**NL4.3/WT**	**NL4.3/DS5000**^**R**^	**NL4.3/AMD3100**^**R**^	**AZT**^**R**^	**NNRTI**^**R**^_**K103N:Y181C**_	**HIV-2 ROD**	
			(165)	(>100)	(>30)	(>85)		
WDO-217	1.04 ± 0.2	0.9 ± 0.05	1.2 ± 0.1	1.7 ± 0.9	1.5 ± 0.6	1.3 ± 0.4	1.04 ± 0.3	75 ± 11

GFP-assay in Jurkat cells.

Values are presented as mean ± std dev from at least 3 independent experiments. Fold resistance of mutant strains towards the respective inhibitor of resistant strains is given in parenthesis (EC_50_ for NL4.3 wild-type as 1).

*EC*_*50*_: 50% effective concentration, concentration of inhibitor required for 50% inhibition of viral replication.

*CC*_*50*_: 50% cytotoxic concentration, concentration of inhibitor that kills 50% of the cells.

### Inactivation of HIV-1, including clinical isolates, and HIV-2 virions by 2-methyl-3-phenyl-2*H*-[1,2,4]thiadiazol-5-ylideneamine

To explore the direct effect of WDO-217 on virus particles, HIV-1III_B_ or HIV-2ROD was incubated for 1 h at 37°C in the presence or absence of different concentrations of the compound. Then the virus was diluted to such extent that the residual compound concentration was far below its antiviral effective concentration, and this diluted virus amount was used to infect MT-4 cell cultures. Virus infectivity was determined by titration and CCID_50_ calculation (Table 4). WDO-217 attenuated both HIV-1 and HIV-2 infectivity, as did the Triton X-100 control, a detergent commonly used in laboratory practice to inactivate HIV. When the virus was incubated at compound concentrations of 26, 130 and 650 μM WDO-217, it was no longer able to infect cells, indicating that these concentrations were able to entirely inactivate the HIV virions. Similar results were obtained with 0.5% Triton-X-100. In contrast, AZT, an inhibitor of the HIV reverse transcription that is inhibiting the viral replication [21], but not directly inactivating HIV virions, was completely devoid of inhibitory activity in this assay. This demonstrates that the compound is sufficiently diluted during the procedure and no significant amount of residual AZT is retained in the sample during titration for CCID_50_ determination.

**Table 4:** Inactivation of isolated HIV particles by WDO-217

**Compound**	**CCID**_**50 **_**/ml**	
	**HIV-1 III**_**B**_	**HIV-2 ROD**
No drug	1 × 10^6^	5.4 × 10^6^
AZT (3.7 μM)	1 × 10^6^	5.4 × 10^6^
Triton X-100 (0.5%)	0	0
WDO-217 (0.2 μM)	5.4 × 10^6^	1.6 × 10^6^
WDO-217 (1 μM)	1.6 × 10^6^	1 × 10^6^
WDO-217 (5 μM)	1 × 10^6^	2.1 × 10^6^
WDO-217 (25 μM)	0	0
WDO-217 (125 μM)	0	0
WDO-217 (625 μM)	0	0

*CCID*_*50*_: 50% cell culture infectious dose as determined by the Reed and Muench method.

The same experiment was repeated for clinical isolates from different HIV-1 subtypes of group M. Virus was incubated in the presence or absence of WDO-217 for 1 hour at 37°C, and virus infectivity was determined by titration on freshly isolated PBMCs from a healthy donor. WDO-217 was able to also inactivate these clinical HIV-1 isolates (Table 5).

**Table 5:** Inactivation of clinical isolates from different HIV-1 subtypes by WDO-217

	**CCID**_**50**_**/ml**				
	**UG275**	**ETH2220**	**UG270**	**BZ163**	**BCF-Dioum**
	**subtype A**	**subtype C**	**subtype D**	**subtype F**	**subtype G**
untreated control	16242	64969	25781	16242	25781
WDO-217 (125 μM)	2558	2558	6446	2558	2558

*CCID*_*50*_: 50% cell culture infectious dose as determined by titration on PBMCs.

### Effect of 2-methyl-3-phenyl-2*H*-[1,2,4]thiadiazol-5-ylideneamine on the capture of HIV-1 by Raji/DC-SIGN cells and on subsequent virus transmission to CD4^+^ T cells

Because WDO-217 has virucidal properties, we next investigated whether it was able to prevent the DC-SIGN-mediated virus capture and subsequent transmission to CD4^+^ T cells, in view of its potential use as a microbicide candidate drug. First, the capacity of WDO-217 to prevent capture of HIV-1 (III_B_) by DC-SIGN was evaluated using Raji/DC-SIGN cells abundantly expressing DC-SIGN in their cell membranes. Exposure of Raji/DC-SIGN cells to HIV-1 captured the virus to their cell membranes, while wild-type Raji/0 cells did not [22]. Virus was pre-exposed to different concentrations of WDO-217 before it was administered to the Raji/DC-SIGN cell cultures. After 60 minutes of pre-incubation with the Raji/DC-SIGN cells, unabsorbed virus and test compound were carefully removed by repeated washing steps, and the amount of captured virus was determined by measurement of the virus-associated p24 content on the Raji/DC-SIGN cell surface. The α(1-3)/(1-6)-mannose-specific plant lectin HHA was included as a positive control for inhibition of virus capture by Raji/DC-SIGN cells. In contrast to HHA, WDO-217 was not able to prevent binding of HIV-1 to the Raji/DC-SIGN cells (Table 6, procedure A), suggesting that the compound does not negatively affect the surface glycoproteins of the virus particles and their function.

**Table 6:** Inhibition of HIV-1 NL4.3 capture and transmission* by Raji/DC-SIGN cells, quantified by p24-ELISA

**Compound**	**IC**_**50 **_**(μM)**		
	**HIV-capture procedure A**	**Transmission procedure B**	**Transmission procedure C**
WDO-217	>105	2.2 ± 1	8.3 ± 0.4
HHA	0.014 ± 0.01	0.002 ± 0.001	≤0.003

*In co-cultivation with C8166 cells; samples examined after 42 hours of incubation.

**Procedure A**: HIV was pretreated (1 h) with compound before it was added to Raji/DC-SIGN cells. Capture was measured by p24 analysis of the washed cell pellet.

**Procedure B**: Raji/DC-SIGN cells were exposed to compound-pretreated (1 h) HIV after which compound was removed and the cells were cocultured in the presence of C8166 cells. The read-out of this experiment was the replication capacity of virus in C8166 as monitored by p24 analysis.

**Procedure C**: HIV-captured Raji/DC-SIGN cells were cocultivated with C8166 cells in the presence of compound (microscopic measurement of giant cell formation).

Next, we evaluated whether WDO-217 could prevent the transmission of captured HIV-1 from DC-SIGN-expressing cells to CD4^+^ T cells. Therefore, the DC-SIGN^+^ cells that efficiently captured drug-treated virus were co-cultured with uninfected C8166 cells. In these co-cultures, WDO-217 dose-dependently inhibited syncytium formation at an IC_50_ of 2.2 μM, whereas abundant syncytium formation occurred within 24 to 48 h post co-cultivation when the captured virus had not been pre-exposed to the drug (Table 6, procedure B). When virus was first given the opportunity to be captured by Raji/DC-SIGN cells in the absence of compound and then the Raji/DC-SIGN cells were co-cultured with C8166 cells in the presence of various concentrations of compound, WDO-217 still prevented the transmission of DC-SIGN-captured virus with an IC_50_ value of 8.3 μM. This suggests that WDO-217 can inactivate virus particles even when they are bound to DC-SIGN (Table 6, procedure C). All together, these results demonstrate that 2-methyl-3-phenyl-2*H*-[1,2,4]thiadiazol-5-ylideneamine does not inhibit the viral capture by DC-SIGN but is able to inactivate captured virus and efficiently prevents the transmission of HIV-1 from DC-SIGN-expressing cells to CD4^+^ T-lymphocytes, underlining its potential use as a microbicide agent.

### Treatment of HIV-1 with WDO-217 decreases the virion-associated viral RNA content

**Figure 3:**
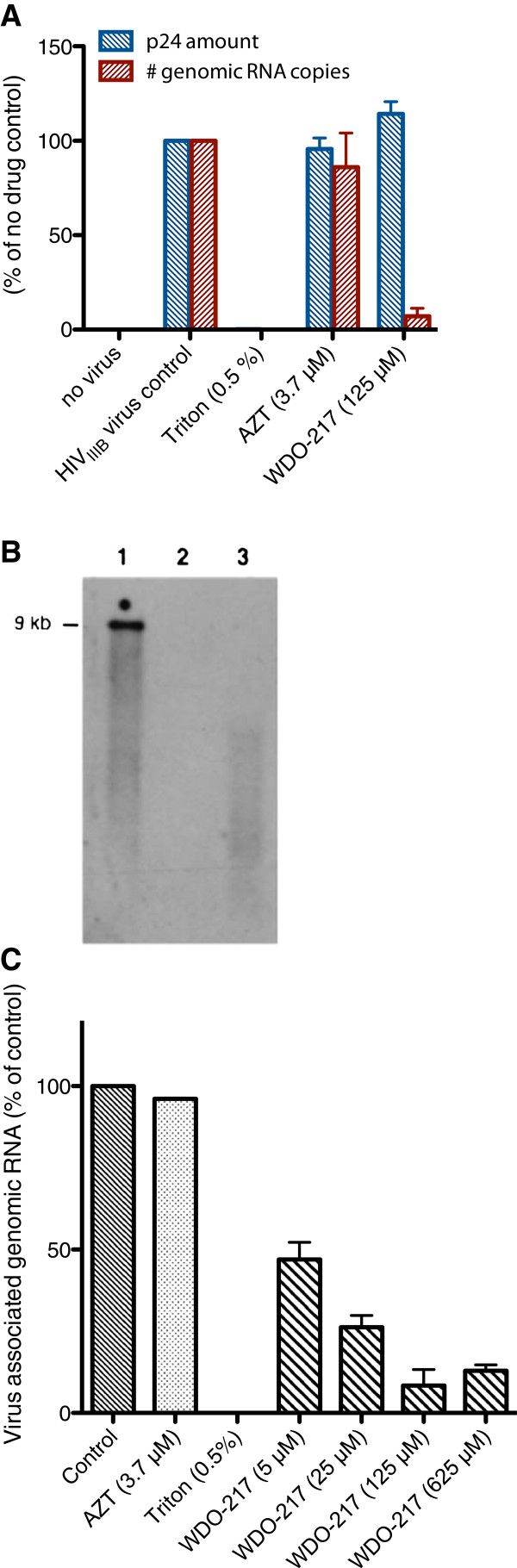
**Effects of WDO-217 on virion-associated Gag p24 CA and genomic mRNA content of HIV-1 virus particles.** Isolated HIV-1 particles were incubated with different concentrations of compounds and subsequently cleared from excess compound by multiple washing and centrifugation steps. (**A**) The amount of virion-associated p24 was quantified by ELISA, and the total virus-associated genomic RNA was quantified by RT-qPCR. (**B**) Genomic RNA of untreated virus (1) or virus treated with 0.5% Triton X-100 (2) or 125 μM WDO-217 (3) was visualized by Northern blot. (**C**) Dose dependent decrease of genomic RNA in treated virus particles as quantified by RT-qPCR. Concentration of compound used is given between brackets. Results are mean ± std dev with n = 3.

To explore the mechanism of HIV inactivation by WDO-217 in more detail, we investigated the effect of WDO-217 on different viral structural components. Therefore, a pre-treated and subsequently compound-cleared virus stock was assessed for both its core p24 antigen protein as well as its viral genomic RNA content (Figure 3). As expected, the detergent Triton-X-100 disturbs the structure of the virions, and no p24 core protein nor viral genomic RNA could be detected in the isolated virus stock after treatment and subsequent washing. In contrast, treatment with WDO-217 did not affect the amount of virus-associated p24 core protein, as for the untreated or AZT-treated control. However, when the viral genomic RNA content of the pre-treated virus stock was quantified by real-time RT-qPCR or visualized by Northern blot, there was a clear decrease in viral RNA for the WDO-treated HIV-1 virions as compared to the untreated control (Figure 3), indicating that WDO-217 reduces the virion-associated RNA stability. Since inside the virion the genomic RNA is protected by the viral nucleocapsid protein (NCp7), this prompted us to investigate the effect of WDO-217 on the nucleocapsid protein.

### Zinc ejection from the retroviral zinc fingers

**Figure 4:**
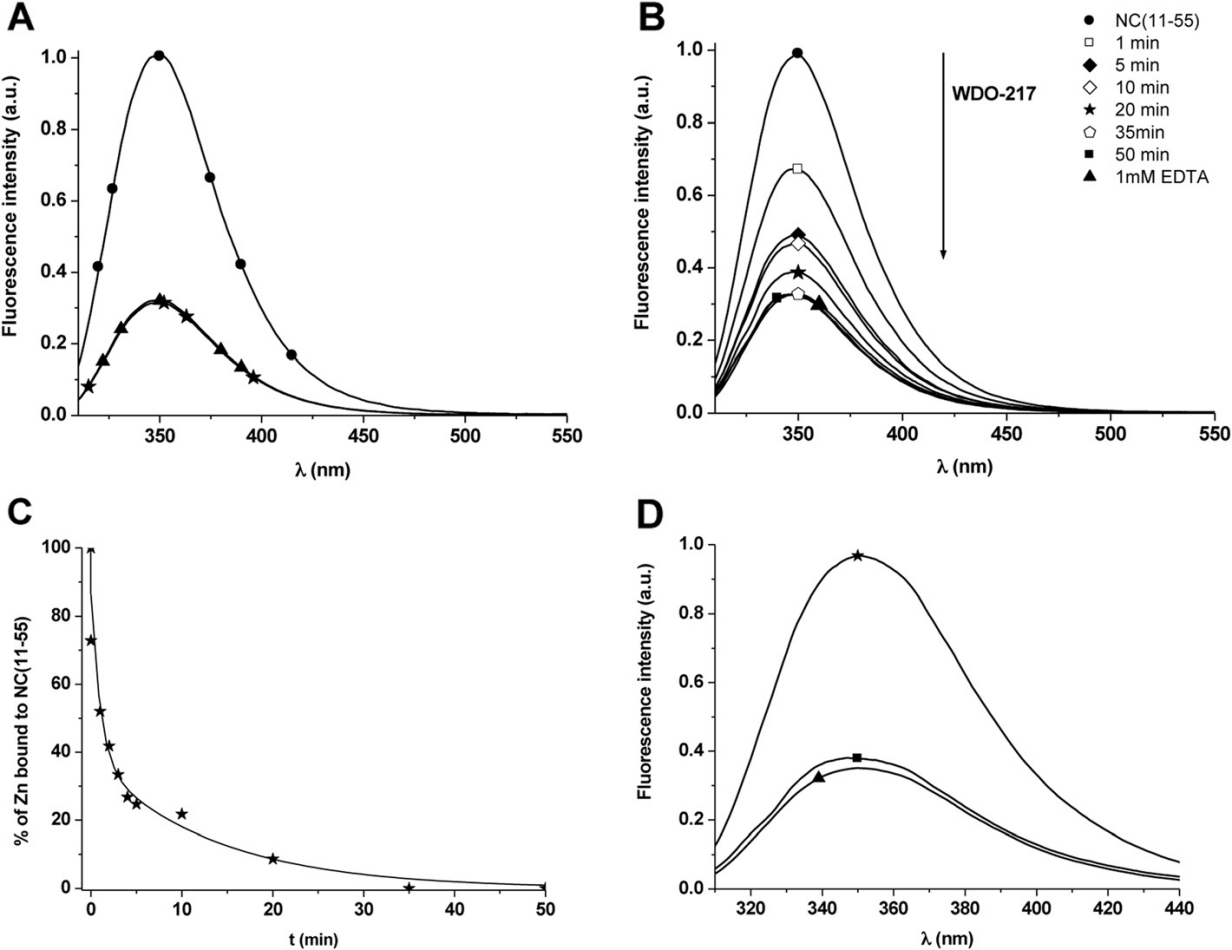
**Zinc ejection from the NCp7(11-55) zinc fingers by WDO-217.** (**A**) Emission spectra of NCp7(11-55) (1 μM) recorded in the absence (disks) and the presence of 10 μM WDO-217 (star). As a reference, NCp7(11-55) was treated with 1 mM EDTA, a well known zinc chelator (triangle). Pre-incubation time of WDO-217 with NCp7(11-55) was 1 hour. (**B**) Time-dependent zinc ejection from NCp7(11-55). The emission spectra of NCp7(11-55) (1 μM) were recorded in the absence (disks) and presence of WDO-217 (10 μM) at the different time points as indicated. (**C**) Kinetics of zinc ejection after addition of 10 μM WDO-217. The data points (stars) corresponded to the fluorescence intensity at the maximum emission wavelength from panel B. Solid line represents a double-exponential fit to the data. (**D**) Zinc ejection from NCp7(11-55) in the presence of an excess of zinc. Emission spectra of the NCp7(11-55) (1 μM) in the presence of an excess of 100 μM zinc (triangle). Then, 10 μM of WDO-217 was added, and the spectrum was recorded after 30 minutes of incubation (square). The emission spectrum of NCp7(11-55) incubated with 1 mM EDTA (triangle) for one hour is given as a reference.

The zinc-ejecting properties from the NCp7 zinc fingers by WDO-217 were investigated by monitoring the intrinsic fluorescence of the Trp37 residue of NCp7(11-55), which shows a 3-fold decrease in its fluorescence quantum yield on zinc removal [23,24]. Addition of a 10-fold excess of WDO-217 (10 μM) induced the same decrease of Trp37 fluorescence as observed for 1 mM EDTA, indicating that WDO-217 efficiently ejects zinc from NCp7(11-55) (Figure 4A). Subsequently, the zinc ejection from NCp7(11-55) by WDO-217 exposure was investigated in function of time (Figure 1B and C). The compound induced a progressive decrease in Trp37 fluorescence and zinc ejection was complete after 35 minutes. Ejection of 50% of the zinc is observed in less than 5 minutes (Figure 4C). A large excess of Zn^2+^ ions (100-fold excess of zinc sulphate) did not prevent the zinc ejecting capacity of WDO-217, since nearly complete ejection of zinc from NCp7(11-55) was observed after 30 minutes (Figure 4D). This result suggests that WDO-217 does not directly chelate zinc ions but progressively ejects the zinc ions from the NCp7 zinc fingers. All together, our results indicate that WDO-217 is a very efficient zinc ejector from NCp7.

**Figure 5:**
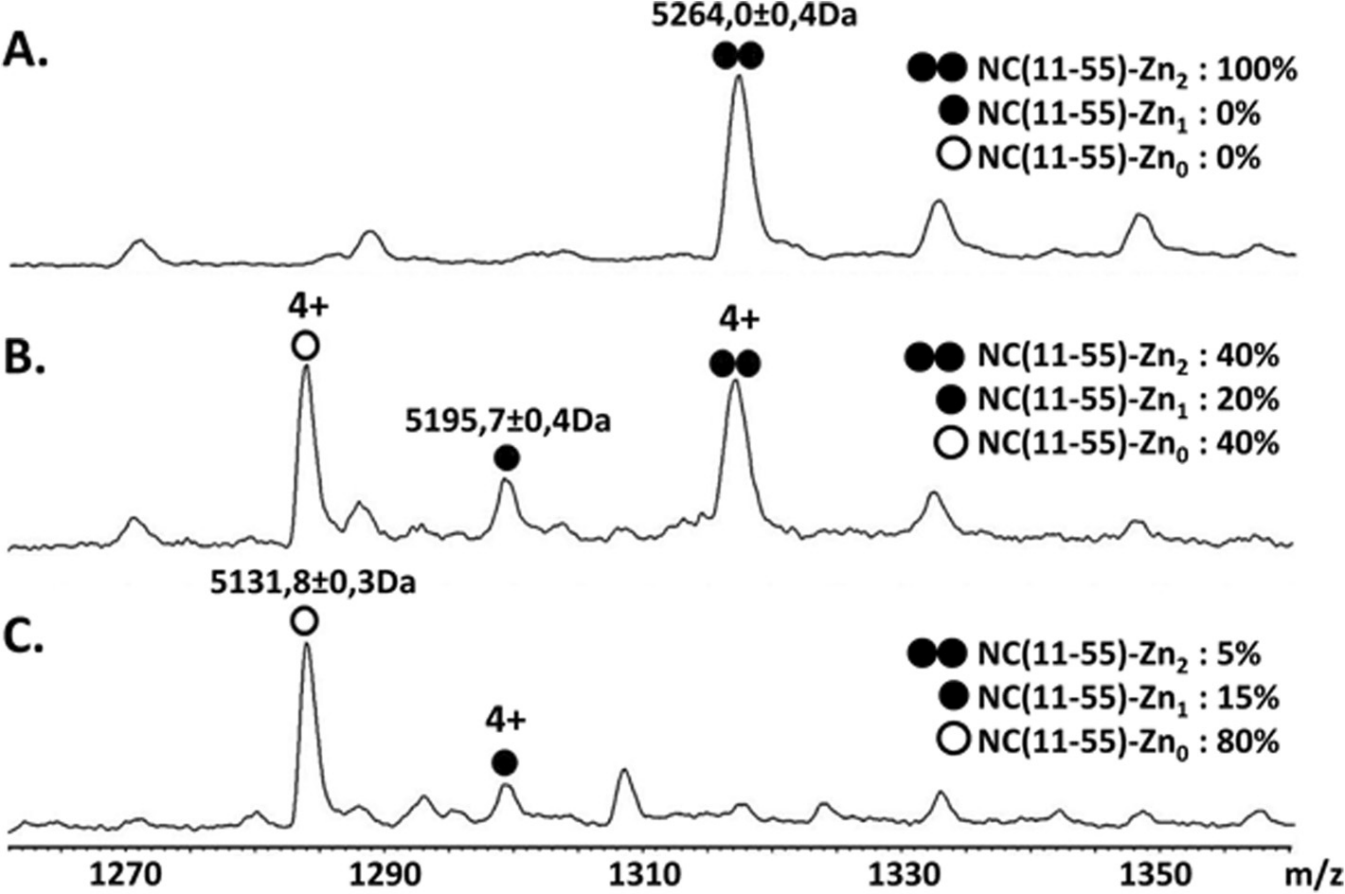
**Zinc ejection as monitored by mass spectrometry.** Supramolecular mass spectrometry analysis on 20 μM NCp7(11-55) (**A**) in the absence of WDO-217 or after 30 min incubation at room temperature with either (**B**) 40 μM WDO-217 or (**C**) 100 μM WDO-217 in 50 mM ammonium acetate, pH7.0. Under non-denaturing conditions (Vc = 20 V, Psi = 6 mbar), the mass measured for NCp7(11-55) alone is 5264.0 Da corresponding to the NC peptide complexed with two zinc ions. Five times molar excess of WDO-217 leads to approximately 80% zinc ejection.

To further investigate the zinc ejection properties of WDO-217, we analyzed the changes in the mass of NC(11-55) by supramolecular mass spectrometry (MS). Purity and homogeneity of NC(11-55) were verified by mass analysis in denaturing conditions. In these acidic conditions, zinc is released from the peptide; and a molecular weight of 5137. 7 ± 0.4 Da was measured, in agreement with the expected mass of the zinc-free NC form (5137.9 Da). In non-denaturing conditions, NC(11-55) analysis revealed the presence of a unique ion series of 5264.0 ± 0.4 Da corresponding to NC(11-55) with 2 zinc ions attached (Figure 5A). Similar MS experiments were then performed in the presence of increasing WDO-217 amounts in order to assess the WDO-127 effect on NC/zinc complexes (Figure 5B and 5C). In the presence of a 2-fold excess of WDO-127, three different peaks corresponding to the native form of NC(11-55) with 2 zinc ions (MW = 5264.0 ± 0.4 Da), NC(11-55) with one zinc ion (MW = 5195.7 ± 0.1 Da) and the zinc free form of NC(11-55) (MW = 5131.8 ± 0.3 Da) were observed, confirming that WDO-217 was able to eject zinc (Figure 5B). Using higher WDO-217 concentrations (five-fold excess of WDO-127 over NC) led to more efficient zinc ejection, as the major species detected (80%) corresponded to the zinc-free form of NC(11-55). Noticeably, zinc ejection leads to the formation of three disulfide bridges as suggested from the differences in molecular weights in denaturing conditions for NC(11-55) in the absence (5137. 4 ± 0.4 Da) and the presence (5131. 4 ± 0.6 Da) of WDO-127. Altogether these MS results unambiguously confirmed that WDO-217 acts as a zinc-ejector. Interestingly, no covalent complex between NC(11-55) and WDO-217 was observed in our experimental conditions (even in very mild MS conditions, Vc = 20 V and Pi = 6 mbar), suggesting that the interaction between the two species is likely transient. This is in contrast to the earlier discovered DIBA zinc ejectors for which the Zn ejection is accompanied by the formation of covalent complexes formed between the compound and Cys residues of Zn-depleted NCp7 [25].

### Inhibition of NCp7(11-55)-induced destabilization of cTAR

**Figure 6:**
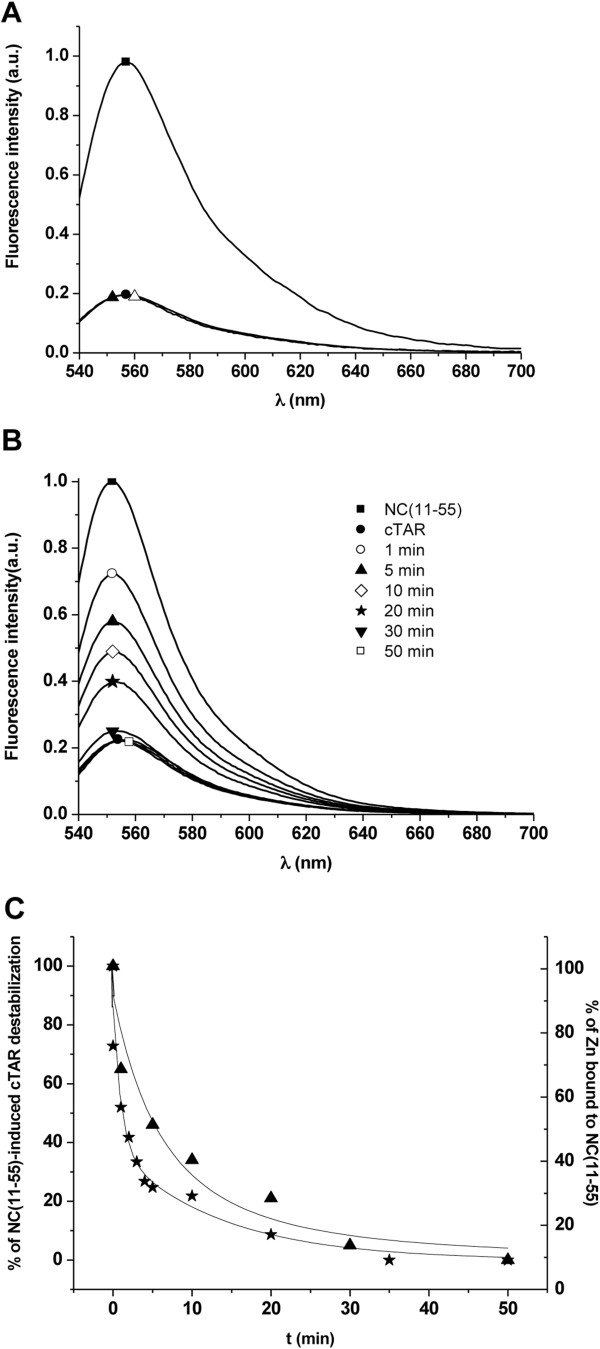
**Inhibition of NCp7(11-55)-induced cTAR destabilization by WDO-217.** (**A**) Emission spectra of Rh6G-cTAR-Dabcyl (0.1 μM) recorded in the absence (circle) or in the presence of NCp7(11-55) (1 μM) (square). To determine the importance of the order of addition of the compounds, the emission spectra of Rh6G-cTAR-Dabcyl (0.1 μM) were recorded either after addition of NCp7(11-55) preincubated with 10 μM WD0-217 (open triangle) or after preincubation with 10 μM WDO-217 and then, addition of 10 μM NCp7(11-55) (closed triangles). Excitation wavelength was 520 nm. (**B**) Inhibition kinetics of NC-induced cTAR destabilization by WDO-217. Emission spectra of Rh6G-cTAR-Dabcyl (0.1 μM) were recorded in the absence (circle) and in the presence of NCp7(11-55) (1 μM) (square) and at different times after addition of WDO-217 (10 μM) as indicated. Excitation wavelength was 520 nm. (**C**) Correlation between the kinetics of Zn^2+^ ejection and inhibition of NC-induced cTAR destabilization by WDO-217 (10 μM). Inhibition of cTAR destabilization (triangle) correlates well with zinc ejection (star).

To confirm the zinc ejection, we measured the ability of NC(11-55) to induce the destabilization of the secondary structure of cTAR DNA in the presence of WDO-217. cTAR is the complementary sequence of the transactivation response element, involved in the minus strand DNA transfer during reverse transcription [26-28]. The NC(11-55)-induced destabilization is exquisitely sensitive to the proper folding of the zinc-bound finger motifs and totally disappears when zinc ions are removed [29]. The NCp7-induced destabilization of cTAR can be sensitively monitored by using the doubly labeled Rh6G-5^′^-cTAR-3^′^-Dabcyl derivative. In the absence of NCp7, cTAR is mainly in a non-fluorescent closed form where the Rh6G and Dabcyl labels, respectively at the 5^′^ and 3^′^ termini of the cTAR stem are in close proximity of each other [30]. As can be noticed from Figure 6A, and in agreement with previous data [30], NC(11-55) added to cTAR at a 10-fold molar excess led to a melting of the bottom of the cTAR stem, which increases the distance between the two fluorophores and thus increases the Rh6G fluorescence. In line with the zinc ejection hypothesis, preincubation of NC(11-55) with WDO-217 at a molar ratio of 10:1 in respect with NC(11-55), led to a full loss of NC(11-55) ability to destabilize cTAR. Interestingly, there was no difference in activity on NCp7-induced cTAR destabilization by WDO-217 when the compound was preincubated first with NCp7 or first with cTAR (Figure 6A). This suggests that WDO-217 can dissociate zinc from NCp7 even when the protein is bound to nucleic acids. The inhibition of the NC(11-55)-induced destabilization of cTAR by WDO-217 was further monitored as a function of time (Figure 6B). A complete inhibition was observed after 50 minutes of incubation with WDO-217, and 50% inhibition was reached in less than 5 minutes in close correlation with its zinc ejection profile (Figure 6D), further confirming the effect of WDO-217 on NC(11-55).

### Effect of WDO-217 on the interaction of NCp7(11-55) with SL3 or PBS

**Figure 7:**
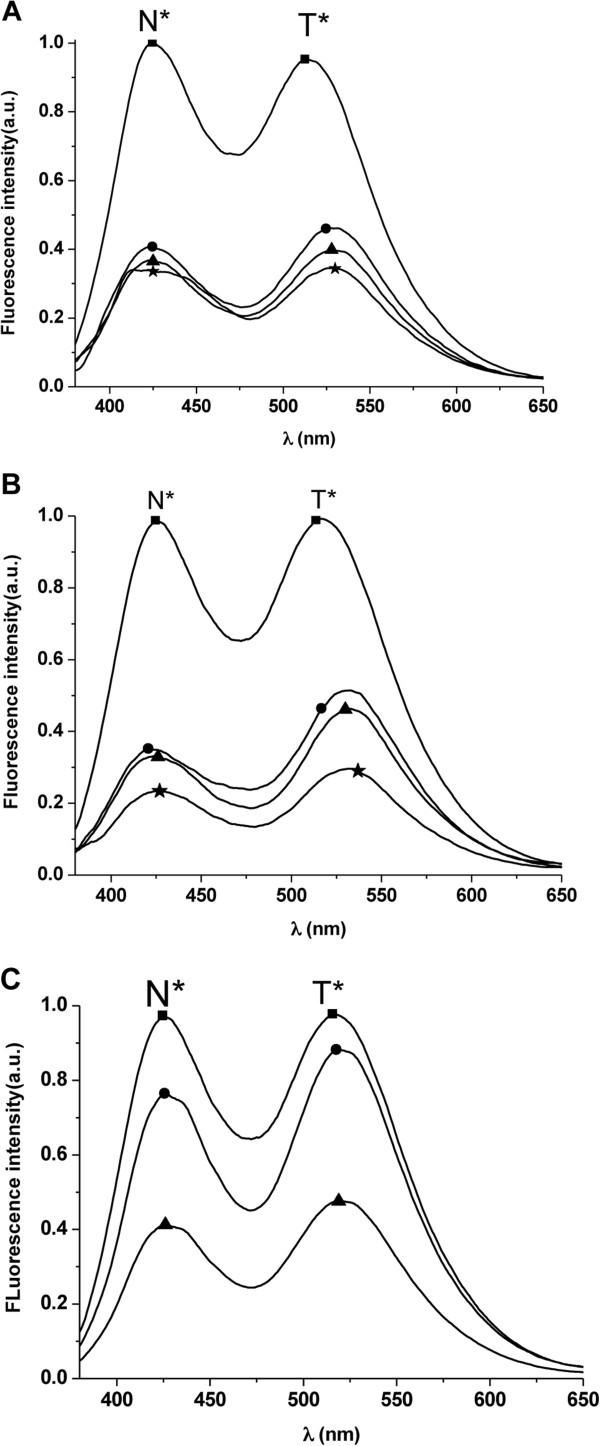
**Effect of WDO-217 on the interaction of NCp7(11-55) with SL3 or PBS.** (**A**) Effect of WDO-217 on the emission spectra of 3HC-NC(11-55) complexed with ΔP(-)PBS. Emission spectra of 3HC-NC (0.2 μM) in the absence (square) and in the presence of ΔP(-)PBS (0.2 μM)(disk). For monitoring the effect of 10 μM WDO-217, the spectra were recorded immediately after its addition to the complex (triangle) and after 30 minutes (star). (**B**) Effect of WDO-217 on the emission spectra of 3HC-NCp7(11-55) complexed with SL3 RNA. Conditions and symbols are as in A. (**C**) Importance of the order of addition of the reactants on the emission spectra of 3HC-NCp7(11-55) complexed with SL3. The emission spectra of 3HC-NC(11-55) (0.2 μM) in the absence (square) and in the presence of WDO-217 (10 μM) (circle). SL3 (0.2 μM) was added to 3HC-NCp7(11-55) (0.2 μM) pre-incubated with WDO-217 (10 μM) for 30 minutes (triangle). Spectra were recorded in 10 mM phosphate buffer, 100 mM NaCl, pH 7.0. Excitation wavelength was 340 nm.

To explore the effect of WDO-217 on the nucleic acid binding properties of NCp7(11-55), we used 3-hydroxyflavone (3HC) labeled NC(11-55) peptide. The 3HC probe shows a two-band emission highly sensitive to the binding of nucleic acids [31]. This two-band emission is the result of a proton transfer reaction that generates two excited states: a normal one (N*) and a tautomeric one (T*). Due to their different dipole moments, these two forms are differently sensitive to the environment. To test the binding properties of 3HC-NCp7(11-55), the SL3 RNA and Δ(-)PBS DNA sequences were selected since they preferentially bind one NCp7 per oligonucleotide (ODN) [32,33]. SL3 corresponds to the third stem-loop sequence of the HIV-1 encapsidation sequence, while Δ(-)PBS DNA corresponds to the (-) Primer Binding Site sequence lacking its 3^′^ single strand overhang. Accordingly, we examined the changes in the emission spectra of 3HC-NCp7(11-55)/oligonucleotide complexes after treatment with WDO-217. Addition of Δ(-)PBS DNA or SL3 RNA to 3HC-NCp7(11-55) was found to decrease the overall intensity of the 3HC probe as well as the N*/T* ratio of its two emission bands in respect to the free 3HC-NCp7(11-55) peptide (Figure 7A and B). This is a result of the stacking of the probe with the bases and its contact with the ODN backbone in the peptide/ODN complexes [31]. Addition of WDO-217 induces a further decrease in the intensity of the spectrum, suggesting that WDO-217 does not dissociate the NCp7/oligonucleotide complex. This additional decrease in fluorescence accompanied by a slight increase in the N*/T* ratio is likely due to a rearrangement of the zinc-free peptide on the ODN sequence, which leads to a change in the interaction of the 3HC probe with the ODN. The increase in the N*/T* ratio observed with the zinc-depleted complex indicates that the environment of the 3HC probe is more polar than in the initial 3HC-NCp7(11-55)/oligonucleotide complex, suggesting a shift from stacking interactions towards more polar interactions with the backbone.

Next, we monitored the changes in the emission spectrum of 3HC-NCp7(11-55) that was preincubated with WDO-217 for 30 minutes, before addition to SL3 (Figure 7C). The incubation of 3HC-NCp7(11-55) with WDO-217 for 30 minutes leads to a decrease in the overall intensity of the 3HC probe as well as its N*/T* ratio (from 1.0 to 0.86) in respect with the free 3HC-NCp7(11-55). This is likely the result of a stronger interaction of the 3HC probe with the peptide backbone when it is in the zinc-free form. Addition of SL3 to the zinc-depleted 3HC-NC(11-55) induced a further decrease of the fluorescence emission resulting in a spectrum similar to that obtained when WDO-217 was added to the preformed 3HC-NC(11-55)/SL3 complex (Figure 7B). This result confirms that WDO-217 is able to eject zinc from the NC/ODN complex.

### WDO-217 does not cause accumulation of unprocessed Gag polyprotein

**Figure 8:**
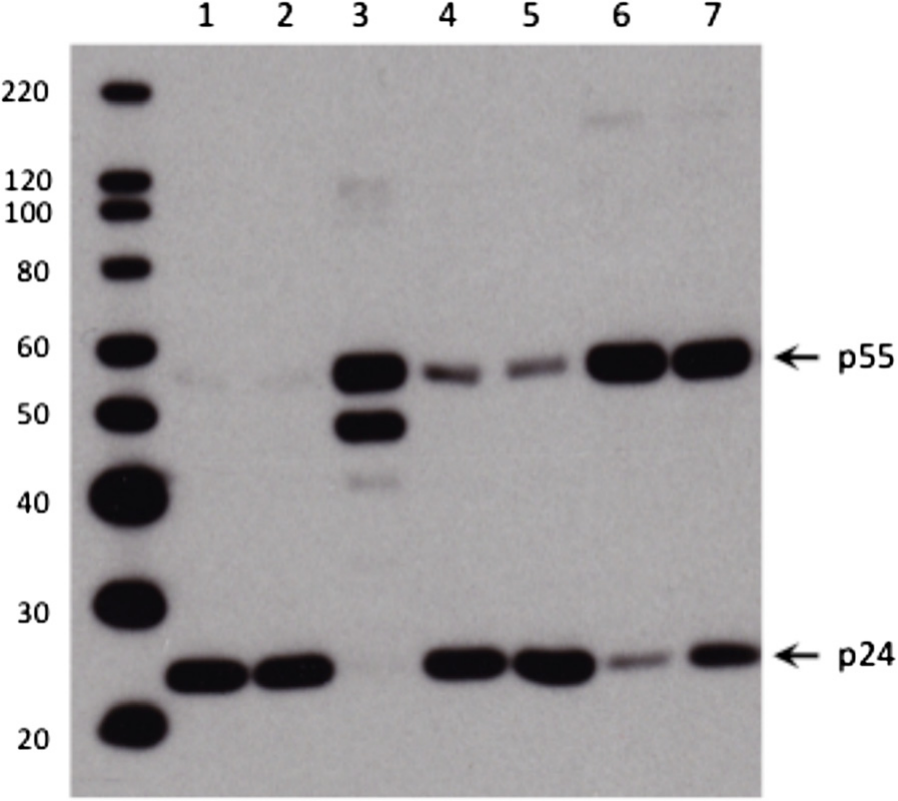
**WDO-217 does not cause accumulation of unprocessed Gag polyprotein.** HIV-1 III_B_ virions from persistently infected HuT-78 cells treated with different concentrations of compound were analysed by gel electrophoresis and immunoblotted with antibody against capsid. 1: untreated; 2: AZT (3.7 μM); 3: ritonavir (2.7 μM); 4: WDO-217 (50 μM); 5: WDO-217 (25 μM); 6: SRR-SB3 (75 μM); 7: SRR-SB3 (15 μM).

When added to infected cells, several NCp7 zinc ejecting compounds, such as SRR-SB3 [34] and SAMTs [35], have been demonstrated to inhibit HIV replication by preventing Gag precursor protein processing and causing accumulation of aggregated, unprocessed Gag polyprotein. Recently, it has been shown that this new mechanism involves acetylation of the NCp7 region of Gag, thereby blocking Gag processing [35]. We investigated whether WDO-217 was able to induce a similar effect when added to virus-infected cells. For this experiment, HIV-1 III_B_ chronically-infected HuT-78 cells were treated with different WDO-217 concentrations. Analysis of the progeny virus in the supernatant demonstrated that treatment with WDO-217 did not result in a drastic accumulation of unprocessed Gag polyprotein (Figure 8). As controls, the protease inhibitor ritonavir and the zinc ejector SRR-SB3 did effectively increase the appearance of unprocessed Gag.

## Conclusion

The retroviral zinc fingers of the HIV-1 NCp7 are strictly conserved and functionally obligatory in the viral replication and virion stability. In contrast to the zinc fingers, the N-terminal domain is less conserved, but the positions of the basic residues are mainly conserved; also the basic linker between the two zinc fingers is highly conserved [36,37]. Early studies have demonstrated that disruption of the zinc fingers by removal of the zinc led to a loss of viral replication [38]. Different classes of zinc ejectors have been reported to efficiently eject zinc from the NCp7 zinc fingers. More specifically, the N-substituted S-acyl-2-mercaptobenzamides (SAMTs) are suggested as candidate pluripotent, HIV-specific, virucidal microbicides [16]. Such nucleocapsid inhibitors were directly virucidal by preventing the initiation of reverse transcription and modifying the CCHC amino acid domain conformation within Gag [11,39]. Here, we have identified 2-methyl-3-phenyl-2*H*-[1,2,4]thiadiazol-5-ylideneamine (WDO-217) as a very potent ejector of zinc ions from the HIV-1 NCp7 zinc fingers, inactivating HIV-1 and HIV-2 virions and relieving the protection of the viral RNA by the retroviral nucleocapsid protein. The exact detailed mechanism by which the RNA is degraded is currently under study. Interestingly, WDO-217 is able to dissociate zinc ions from NCp7 even when it is bound to nucleic acids. In agreement with the comparable affinities of the native and zinc-depleted NCp7 forms for oligonucleotides [29,40,41], ejection of zinc from NCp7 by WDO-217 should not dissociate the protein from the oligonucleotide. However, a change in the binding mode is expected since native NCp7 was shown to strongly interact with the bases of its nucleic acid targets through van der Waals, H-bonding and stacking interactions [32,33,42-44], while the zinc-depleted forms of NCp7 are thought to interact mainly through electrostatic interactions with the phosphate groups of the DNA or RNA backbone [45,46]. The differences in the binding modes of the native and zinc-depleted forms have been clearly evidenced by the fact that only the former can modify the dynamics of the bases and structurally rearrange the oligonucleotides [32,45,46]. In contrast to the DIBA compounds [25] WDO-217 is found not to form a covalent complex with NCp7. This could be of interest because it would in principle allow using a lower concentration of compound as one molecule can react with more than one NCp7 as compared to compounds that react covalently. A disadvantage of a non-covalent binder could be that zinc ejection may be reversible if it is not followed by oxidation of the cysteines in the zinc fingers. As a consequence, the concentration of WDO-217 should be high enough to maintain an efficient depletion of zinc, so that oxidation of cysteines will occur. Although its antiviral selectivity is somewhat moderate, WDO-217 represents the first lead compound among zinc-ejecting compounds with a unique mode of action. Due to its moderate therapeutic index this lead compound would be more appropriate as microbicide then as a systemic therapeutic agent. Future structure activity relationship studies and molecular modeling studies, however, will likely enable improvement on activity, selectivity and pharmacological properties of the compound. Although WDO-217, as such, is a simple molecule there is room for further modifications and subsequent SAR studies. Modifications are possible introducing substituents at different positions on the phenyl ring. It is certainly worthwhile to further investigate the importance of the relative positions and nature of the substituents on the 1,2,4-thiadiazol ring. Furthermore it would be interesting switching to other heterocyclic rings, the most obvious being a series of 4,5-disubstituted -2-aminothiazole derivatives.

Interestingly, WDO-217 treatment does not inhibit the direct gp120-mediated capture of virus on DC-SIGN suggesting that it is able to inactivate HIV but with a preservation of the conformational and functional integrity of the surface envelope proteins. This preservation of surface proteins has earlier been demonstrated with the prototypical NCp7 zinc ejector AT-2 [47]. Moreover, WDO-217 inactivates virus particles even when they are bound to DC-SIGN and prevents the transmission of DC-SIGN-captured HIV to CD4^+^ cells. This makes WDO-217 suitable, not only as a valuable component in topical microbicides, but also for the inactivation of virions to be used as vaccine antigens [47]. Indeed, HIV vaccine strategies using DCs are currently being investigated. DCs detect viruses in peripheral tissues and, following activation and virus capture/uptake, migrate to lymph nodes to trigger adaptive immune responses [48]. However, HIV is able to modulate these DCs to facilitate infection and transmission to T-cells causing their disregulation [49]. This justifies the need to develop strategies that prevent this modulation and disregulation. In this respect, WDO-217 appears of high interest, since it does not prevent the capture by DC SIGN but prevents subsequent transmission and infection.

This new compound is useful as a lead for the design of a future new generation NCp7 inhibitors that could be applied as an agent in microbicide formulations and vaccine strategies.

## Methods

### Cells and viruses

MT-4, Jurkat A72, CEM, HuT-78 and Raji/DC-SIGN cells were grown and maintained in RPMI 1640 supplemented with 10% heat-inactivated fetal calf serum, 2 mM L-glutamine, 0.1% sodium bicarbonate and 20 μg gentamicin per ml. The HIV-1(III_B_) strain was provided by R.C. Gallo and M. Popovic (at that time at the NIH, Bethesda, MD, USA). HIV-2(ROD) was obtained from L. Montagnier (at that time at the Pasteur Institute, Paris, France) and SIV(Mac251) from C. Bruck. Raji/DC-SIGN were kindly provided by L. Burleigh (Paris, France).

### *In vitro* antiviral assays

Evaluation of the antiviral activity of the compounds against HIV-1 strain III_B_ in MT-4 cells was performed using the MTT assay as previously described [18,19]. Stock solutions (10 x final concentration) of test compounds were added in 25 μl volumes to two series of triplicate wells so as to allow simultaneous evaluation of their effects on mock- and HIV-infected cells at the beginning of each experiment. Serial 5-fold dilutions of test compounds were made directly in flat-bottomed 96-well microtiter trays using a Biomek 3000 robot (Beckman instruments, Fullerton, CA). Untreated HIV- and mock-infected cell samples were included as controls. HIV-1(III_B_) stock (50 μl) at 100-300 CCID_50_ (50% cell culture infectious doses) or culture medium was added to either the infected or mock-infected wells of the microtiter tray. Mock-infected cells were used to evaluate the effects of test compound on uninfected cells in order to assess the cytotoxicity of the test compounds. Exponentially growing MT-4 cells were centrifuged for 5 minutes at 220 g, and the supernatant was discarded. The MT-4 cells were resuspended at 6 x 10^5^ cells/ml and 50 μl volumes were transferred to the microtiter tray wells. Five days after infection, the viability of mock-and HIV-infected cells was examined spectrophotometrically using the MTT assay. The MTT assay is based on the reduction of yellow coloured 3-(4,5-dimethylthiazol-2-yl)-2,5-diphenyltetrazolium bromide (MTT) (Acros Organics) by mitochondrial dehydrogenase activity in metabolically active cells to a blue-purple formazan that can be measured spectrophotometrically. The absorbances were read in an eight-channel computer-controlled photometer (Infinite M1000, Tecan), at two wavelengths (540 and 690 nm). All data were calculated using the median absorbance value of three wells. The 50% cytotoxic concentration (CC_50_) was defined as the concentration of the test compound that reduced the absorbance (OD_540_) of the mock-infected control sample by 50%. The concentration achieving 50% protection against the cytopathic effect of the virus in infected cells was defined as the 50% effective concentration (EC_50_).

The antiviral activity of the compounds against HIV was evaluated in Jurkat cells stably transformed to express the LTR-GFP (A72 cells) [20,50]. In 96-well plates, 3 × 10^4^ A72 cells were infected with HIV in the presence of various concentrations of test compound. Three days post infection, cells were harvested and fixed in 3% paraformaldehyde. GFP-expression was quantified on a single cell basis by flow cytometry [51,52]. Toxicity of the compounds was tested using an MTT-based method.

HIV-1 core antigen (p24 Ag) in the supernatant was analyzed by the p24 Ag enzyme-linked immunosorbent assay (Perkin Elmer).

### Virucidal assay

Aliquots of a HIV stock (III_B_ or ROD) were incubated with various concentrations of compound in a final volume of 100 μl RPMI-1640 culture medium with 10% FCS for 1 hour at 37°C. For the clinical isolates of different clades, stock was incubated with 125 μM WDO-217 for 1 hour at 37°C. Subsequently, the samples were diluted 4000 times with complete medium so that the residual concentration of compound present was far below its IC_50_. The drug-treated and diluted virus suspension was then used to infect susceptible MT-4 T-cells to quantify the viral infectivity by titration and CCID_50_ calculation [53]. The different clinical isolates were titrated on freshly isolated PBMCs from a healthy donor. Control experiments with AZT indicated that this procedure effectively diluted the compound to concentrations well below its effective antiviral concentration.

### Inhibitory effect on virus production from HuT-78/III_B_ persistently infected cells, virion analysis and western blot

Chronically-infected HIV-1 IIIB HuT-78 cells (HuT-78/III_B_) were washed four times with PBS to remove all free virions before treatment and 2 × 10^5^ cells were resuspended in 1 ml compound-containing medium for 43 hours at 37°C. Then, virions were prepared from clarified supernatants (10 min at 300 g) by centrifugation at 36670 g for 2 hours at 4°C. Protein from lysed virions were separated by SDS-PAGE on a NuPage® Novex 4-12% Bis-Tris gel (Invitrogen) and transferred on a hydrophobic polyvinylidene difluoride (PVDF) membrane (Amersham Hybond™-P). The blot was blocked overnight at 4°C by 5% dry milk powder in Western blot wash solution (WBWS; PBS + 0.5% Tween 20), washed three times for 5 minutes with WBWS and incubated with a mouse anti-HIV-1 p24 antibody (1:5000) from Abcam. The blot was then washed three times for 5 minutes with WBWS and incubated for 1 h with a goat anti-mouse IgG-HRP secondary antibody (1:2500) from Santa Cruz Biotechnology. The blot was washed three times for 5 minutes with WBWS and after 5 minutes of incubation with SuperSignal West Pico Chemiluminescent Substrate (Thermo Scientific) it was developed.

### Effect of WDO-217 on the exposure of HIV-1 to Raji/DC-SIGN cells

In a first set of experiments (procedure A), HIV-1 (NL4.3) was exposed to WDO-217 at 105 μM (in 0.5 ml culture medium) for 60 minutes at 37°C. Then, 0.5 ml exponentially growing Raji/DC-SIGN cells (10^6^ cells) was added, and the suspension further incubated at 37°C for 60 minutes. Subsequently, 39 ml medium was added and the cell suspension was centrifuged at 300 g for 10 minutes. The obtained pellet was washed again with 40 ml medium and after centrifugation, the cell pellet (containing DC-SIGN-bound virus) was analyzed for p24 antigen content by ELISA. In a second set of experiments (procedure B), Raji/DC-SIGN cells treated as in procedure A were co-cultured in the presence of an equal amount of C8166 cells (10^6^ cells) (total volume of 1 ml). Replication in C8166 was measured after ~ 20 hr of incubation. In a third set of experiments (procedure C), Raji/DC-SIGN cells were given the opportunity to capture HIV-1/NL4.3 by mixing Raji/DC-SIGN cells with virus (10^6^ cells/ml). After one hour of incubation at 37°C, C8166 cells (10^6^ cells/ml) were added in the presence of WDO-217 at different concentrations and giant cell formation in the cultures was examined microscopically after approximately 24 hr. In the above-described experiments, the mannose-binding entry inhibitor HHA was included as a reference compound.

### Quantitative RT-PCR

Total mRNA from virus stock was extracted using the QIamp viral RNA kit (Qiagen) followed by DNA digestion using RNase-free DNase I (Invitrogen). DNase I treated mRNA was used to generate cDNA along with Thermoscript reverse transcriptase (Invitrogen) and oligo(dT)_20_. qRT-PCR for genomic unspliced HIV mRNA was performed according to a protocol described earlier [54], using 0.2 mM primers TCAGCCCAGAAGTAATACCCATGT and TGCTATGTCAGTTCCCCTTGGTTCTCT, and 0.2 mM FAM-BHQ1 fluorescent probe ATTAACAGAAGGAGCCACCCCACAAGA. Control reactions omitted reverse transcriptase, and the number of cDNA copies was determined using a HIV-1_NL4.3_ molecular clone DNA standard.

### Zinc ejection and inhibition of NC(11-55)-induced destabilization of cTAR monitored by fluorescence techniques

The NC(11-55) peptide was synthesized by solid phase peptide synthesis on a 433A synthesizer (ABI, Foster City, CA), as previously described [31]. The lyophilized peptide was dissolved in water, and its concentration was determined using an extinction coefficient of 5,700 M^–1^ x cm^–1^ at 280 nm. Next, 2.5 molar equivalents of ZnSO_4_ were added to the peptide and pH was raised to its final value, by adding buffer. The increase of pH was done only after zinc addition to avoid oxidization of the zinc-free peptide. Zinc ejection was monitored through the changes in the intrinsic fluorescence of the Trp37 residue of NC(11-55) [23,24], after addition of a 10-fold excess of WDO-217 (10 μM) to 1 μM NC(11-55).

To monitor the inhibition by WDO-217 of the NC(11-55)-induced destabilization of cTAR, we used doubly labelled cTAR, synthesized at a 0.2 μmol scale by IBA GmbH Nucleic Acids Product Supply (Göttingen, Germany). The 5^′^ terminus of cTAR was labeled with 6-carboxyrhodamine (Rh6G) via an amino-linker with a six carbon spacer arm. The 3^′^ terminus of cTAR was labeled with 4-(4^′^-dimethylaminophenylazo)benzoic acid (Dabcyl) using a special solid support with the dye already attached. The doubly labeled cTAR was purified by reverse-phase HPLC and polyacrylamide gel electrophoresis. An extinction coefficient at 260 nm of 521,910 M^–1^ x cm^–1^ was used for cTAR. All experiments were performed at 20°C in 25 mM Tris–HCl, pH 7.5, 30 mM NaCl, and 0.2 mM MgCl_2_[28]. The effect of WDO-217 on the NC(11-55)-induced destabilization of cTAR was observed after addition of 10 μM WDO-217 to 0.1 μM Rh6G-cTAR-Dabcyl preincubated with 1 μM NC(11-55).

Absorption spectra were recorded on a Cary 400 spectrophotometer. Fluorescence spectra were recorded at 20°C on a Fluorolog spectrofluorometer (Horiba, Jobin-Yvon), equipped with a thermostated cell compartment. Excitation wavelength was 295 nm and 520 nm, for NC(11-55) and Rh6G-5^′^-cTAR-3^′^-Dabcyl, respectively. The spectra were corrected for dilution and buffer fluorescence. The protein spectra were additionally corrected for screening effects due to the zinc ejecting agent, using:

(1) $$I_{P}\;=\;I_{m}\;*\;\frac{\left(d_{p}\;+\;d_{s}\;+\;d_{r}/2\right)\;\left(1-10^{-d_{p}}\right)}{d_{p}\left(1-10^{-\left(d_{p}+d_{s}+d_{r}/2\right)}\right)}$$

where I_m_ is the measured fluorescence of the protein, I_p_ is the fluorescence intensity of the protein in the absence of inner filter, d_p_ is the absorbance of the protein, d_s_ is the absorbance of WDO-217 at the excitation wavelength, and d_r_ is the absorbance of WDO-217 at the emission wavelengths.

### Zinc ejection monitored by supramolecular mass spectrometry

Before mass spectrometry (MS) analysis, NC(11-55) was dissolved and buffer exchanged with 50 mM ammonium acetate pH 7.0 using 4 cycles of microcentrifuge size exclusion filtering (Vivaspin 500 5kD, Sartorius Stedim biotech, Aubagne, France) and peptide concentration was measured by a Bradford assay.

ESI-MS measurements were performed in the positive ion mode on an electrospray time-of-flight mass spectrometer (LCT, Waters, Manchester, UK) equipped with an automated chip-based nanoESI source (Triversa Nanomate, Advion Biosciences, Ithaca, NY). Calibration of the instrument was performed using multiply charged ions of a 2 μM horse heart myoglobine solution. For analysis in denaturing conditions, samples were diluted to 2 μM in a 1/1 water/acetonitrile mixture (v/v) acidified with 1% formic acid and standard interface parameters were used to obtain best mass accuracy. In these conditions, noncovalent interactions are disrupted, allowing the measurement of the molecular weight of the monomer with a good accuracy (better than 0.01%).

Analyses under non-denaturing conditions were carried out after careful optimization of instrumental settings to avoid dissociation of noncovalent bonds and obtain sensible detection of protein/zinc complexation states. The accelerating voltage (Vc) was fixed to 20 V, and the pressure in the first pumping stage of the instrument (Pi) to 6 mbar. Zinc ejection measurements were performed after 30 minutes incubation at room temperature of a 20 μM solution of NC(11-55) with either 40 μM or 100 μM WDO-217. Data analysis was performed with the MassLynx 3.5 software (Waters, Manchester, UK). Peak intensities were used to estimate the ratios of the different ions detected.

## Competing interest

All authors declare that they have no competing interests.

## Authors’ contributions

TV, BB, CP, YM, DD participated in research design; TV, BB, NH, FD, SSC conducted experiments; WD synthesized the lead compound; TV, BB, NH, CP, SSC, JB, YM, DD performed data analysis; DD and YM co-wrote the manuscript. All authors read and approved the final manuscript.
